# Health service use for young males and females with a mental disorder is higher than their peers in a population-level matched cohort

**DOI:** 10.1186/s12913-022-08789-3

**Published:** 2022-11-16

**Authors:** Rebecca J Mitchell, Anne McMaugh, Reidar P Lystad, Cate M Cameron, Olav Nielssen

**Affiliations:** 1 Australian Institute of Health Innovation, Faculty of Medicine, Health and Human Sciences, Macquarie University, Level 6, 75 Talavera Road, NSW 2109 Sydney, Australia; 2 The Macquarie School of Education, Macquarie University, Sydney, Australia; 3 Jamieson Trauma Institute, Royal Brisbane & Women’s Hospital, Metro North Health, Brisbane, Australia; 4 AusHSI, Centre for Healthcare Transformation, Queensland University of Technology (QUT), Brisbane, Australia; 5 Faculty of Medicine, Health and Human Sciences, Macquarie University, Sydney, Australia

**Keywords:** Mental disorders, Hospitalisation, Outpatient, Youth

## Abstract

**Background:**

To inform healthcare planning and resourcing, population-level information is required on the use of health services among young people with a mental disorder. This study aims to identify the health service use associated with mental disorders among young people using a population-level matched cohort.

**Method:**

A population-based matched case-comparison retrospective cohort study of young people aged ≤ 18 years hospitalised for a mental disorder during 2005–2018 in New South Wales, Australia was conducted using linked birth, health, and mortality records. The comparison cohort was matched on age, sex and residential postcode. Adjusted rate ratios (ARR) were calculated for key demographics and mental disorder type by sex.

**Results:**

Emergency department visits, hospital admissions and ambulatory mental health service contacts were all higher for males and females with a mental disorder than matched peers. Further hospitalisation risk was over 10-fold higher for males with psychotic (ARR 13.69; 95%CI 8.95–20.94) and anxiety (ARR 11.44; 95%CI 8.70-15.04) disorders, and for both males and females with cognitive and behavioural delays (ARR 10.79; 95%CI 9.30-12.53 and ARR 14.62; 95%CI 11.20-19.08, respectively), intellectual disability (ARR 10.47; 95%CI 8.04–13.64 and ARR 11.35; 95%CI 7.83–16.45, respectively), and mood disorders (ARR 10.23; 95%CI 8.17–12.80 and ARR 10.12; 95%CI 8.58–11.93, respectively) compared to peers.

**Conclusion:**

The high healthcare utilisation of young people with mental disorder supports the need for the development of community and hospital-based services that both prevent unnecessary hospital admissions in childhood and adolescence that can potentially reduce the burden and loss arising from mental disorders in adult life.

**Supplementary Information:**

The online version contains supplementary material available at 10.1186/s12913-022-08789-3.

## Background

Worldwide an estimated 14% of young people aged 10–19 years experience a mental disorder [1]. Among young people, depression, anxiety, and conduct disorders are the most prevalent mental disorders [1]. In the United States (US), approximately 20% of children and adolescents are living with a mental disorder, with yearly inpatient and outpatient healthcare costs of US$247 billion [2, 3]. In the United Kingdom (UK), around 13% of young people aged 5 to 19 years have a mental disorder [4], and in Australia, an estimated 14% of young people experience a mental disorder [5].

Young people living with a mental disorder experience worse overall physical health, longer lengths of stay (LOS) in hospital, and receive poorer healthcare quality as a result of both their conditions and their circumstances compared to the general population [6–9]. Not only is the health of young people experiencing mental illness adversely affected, but mental disorders have also been associated with poor academic performance among young people and a failure to complete high school [10–13]. The impact on subsequent academic achievement, employment and earning potential are long lasting, as early high school leavers are more likely to experience unemployment compared to their peers [14].

Healthcare use and associated treatment costs are generally higher for young people living with a mental disorder compared to their peers [15]. However, recent studies to quantify the disparity in the use of health services (i.e. emergency department (ED) visits, hospital admissions and ambulatory services) by young people living with a mental disorder compared to the general population in Australia are lacking. Population-level data on health service among young people with a mental disorder compared to the general population will assist in planning health service resourcing, identifying infrastructure needs, workforce development, and in the understanding of acute health care management and follow-up care for young people with a mental disorder [16, 17]. This study aims to identify the health service use associated with mental disorders among young people by sex using a population-level matched cohort.

## Method

This is a population-level case-comparison retrospective cohort study of young people aged ≤ 18 years hospitalised with a mental disorder in New South Wales (NSW), Australia, using linked birth, health and mortality data collections from 1 to 2005 to 31 December 2018 and the methodology as been described elsewhere [18]. This study represents a snapshot in time of the health service use of young people hospitalised with a mental disorder in one jurisdiction.

### Data sources

Information on health service use was obtained from ED visit and hospital admission data collections in NSW. ED visits to public hospitals included data on arrival and departure times, visit type, and provisional diagnosis. Hospital admissions were to public or private hospitals, and contained information on demographics, diagnoses, separation type (e.g. hospital transfer, death), and clinical procedures. Health service use was followed until 30 June 2019. Information on the number and type of ambulatory specialist mental health service visits at public hospitals was obtained from the ambulatory mental health client contacts database from 1 to 2006 to 30 September 2019. This included mental health day programs, public psychiatric outpatients and outreach services. Mortality data was obtained from the NSW Registry of Births, Deaths and Marriages and included date of death. Young people who died during the study timeframe were excluded from the analysis of health service use [18].

The Centre for Health Record Linkage (CHeReL) linked the birth, health and mortality records using probabilistic record linkage. Upper and lower probability cut-offs for a link were 0.75 and 0.25 and record groups with probabilities between the cut-offs were clerically reviewed. The CHeReL also identified the population comparison group [18].

### Case inclusion criteria

Cases included young people with a year of birth ≥ 1997 who were aged ≤ 18 years at their index hospitalisation during 1 January 2005 to 31 December 2018 who had a principal or additional diagnosis (up to 50 additional diagnoses) of a mental disorder identified using the International Classification of Diseases, 10th Revision, Australian Modification (ICD-10-AM) and categorised as: substance disorders (ICD-10-AM: F10-F19), psychotic disorders (ICD-10-AM: F20-F29), mood disorders (ICD-10-AM: F30-F39), anxiety disorders (ICD-10-AM: F40-F48), eating disorders (ICD-10-AM: F50), intellectual disability (ICD-10-AM: F70-79), autism spectrum disorders (ASD) (ICD-10-AM: F84), cognitive and behavioural delay (ICD-10-AM: F80-F83 and F88-F89), and conduct disorders (ICD-10-AM: F90-F98) (Supplementary Table 1). The number of co-occurring mental disorders experienced by the young person was categorised as 1 or ≥ 2 disorders [18]. The principal diagnosis of the first readmission for cases was identified using ICD-10-AM Chapter categories.

### Population-comparison criteria

A population-based comparison group not hospitalised with a mental disorder from 1 to 2001 to 31 December 2018 was randomly selected from NSW birth records matched 1:1 on age, sex and residential postcode to their counterpart. The selection timeframe for comparisons included a 3.5-year wash-out period prior to the case selection timeframe to avoid the potential selection of comparison group members who may have been hospitalised with a mental disorder prior to the case criteria timeframe [18].

### Identification of other health conditions

Other common chronic health conditions for young people were identified from prior studies of paediatric comorbidities [19–21] and were conditions reasonably expected to last 12 months or need ongoing healthcare [19]. For this study, a chronic health condition was identified using a three-year look-back period (to 1 January 2002) and hospital diagnoses classified using ICD-10-AM, excluding the mental disorder of interest (Supplementary Table 2) [18].

### Socioeconomic status and geographical location

The young person’s postcode of residence was used to partition socioeconomic disadvantage into quintiles from most (i.e. 1) to least (i.e. 5) disadvantaged [22]. The quintiles are derived using information such as income, education, employment, and occupation from Australia’s population census. The Australian Statistical Geographical Standard [23] is based on distance to service centres and was used to classify the postcode of residence of the young person as either urban (i.e. major cities) or rural (i.e. inner and outer regional, remote, and very remote) [18].

### Ambulatory mental health client contacts, ED visits, hospital admissions, and hospital length of stay

The number of ambulatory mental health client contacts, ED visits and hospital admissions post the index hospitalisation of the case were identified for both the cases and their matched peers. The calculation of hospital LOS after the index admission was cumulative and included transfers between hospitals. The index admission was not included in the count of ED visits, hospital admissions or in the calculation of cumulative hospital LOS for cases [18].

### Data management and analysis

Data analysis was conducted using SAS 9.4 (SAS Institute, Cary NC). All hospital episodes of care related to the same event were linked to form a period of care. Chi-square tests of independence and Wilcoxon Mann-Whitney tests, as appropriate, were used to examine characteristics of young people hospitalised with a mental disorder and their matched counterpart.

Negative binomial regression, adjusted for mental disorder status, sex, age group, comorbidities (i.e. Y/N), geographic location of residence, and socioeconomic status, with the log of the length of exposure post the index case admission used as an offset, was used to quantify associations between each mental disorder and counts of hospital admissions up to 30 June 2019 using rate ratios and 95% confidence intervals (CI). Matching variables were included in the model to control for any possible confounding from the matching variables [24]. Where rate ratios were calculated by sex or age group, these variables were not included as predictors in the models.

## Results

There were 27,801 young people aged ≤ 18 years hospitalised with a mental disorder during 2005–2018. Of these, 14,143 (50.9%) were male and 13,658 (49.1%) were female. Over half (56.2%) the young people hospitalised were aged ≥ 10 years, with 31.9% aged 15–18 years. Around one-third (31.3%) of young males were aged ≤ 4 years at their index admission compared to 19.6% of females, whereas at 15–18 years females (40.7%) had one and a half times the proportion of hospital admissions compared to males (23.4%).

Almost three-quarters (72.2%) of young people hospitalised for a mental disorder lived in urban areas and across a range of socioeconomic areas. The majority (92.9%) of young people with a mental disorder did not have other chronic health conditions, but they had a higher proportion of other comorbidities compared to their matched peers for both males (8.2% vs. 1.3%, respectively) and females (6.0% vs. 1.6%, respectively). Females (14.3%) with a mental disorder had a higher proportion of co-occurring mental disorders than males with a mental disorder (10.2%) (Table 1).

**Table 1:** Demographic characteristics at the index admission for young people hospitalised with a mental disorder and their matched comparison by sex, linked health and mortality data NSW, 2005–2018

	All persons					Male					Female				
	**Case** (*n *= 27,801)		**Comparison** (*n* = 27,801)			**Case** (*n* = 14,143)		**Comparison** (*n *= 14,143)			**Case** (*n* = 13,658)		**Comparison** (*n* = 13,658)		
**Characteristics**	**n**	**%**	**n**	**%**	***p*-value**	**n**	**%**	**n**	**%**	***p*-value**	**n**	**%**	**n**	**%**	***p*-value**
**Age group at index admission of case**															
0–4	7,093	25.5	7,093	25.5	1.0	4,422	31.3	4,422	31.3	1.0	2,671	19.6	2,671	19.6	1.0
5–9	5,090	18.3	5,090	18.3		3,547	25.1	3,547	25.1		1,543	11.3	1,543	11.3	
10–14	6,752	24.3	6,752	24.3		2,869	20.3	2,869	20.3		3,883	28.4	3,883	28.4	
15–18	8,866	31.9	8,866	31.9		3,305	23.4	3,305	23.4		5,561	40.7	5,561	40.7	
**Location of residence**															
Urban	20,064	72.2	20,064	72.2	1.0	10,360	73.3	10,360	73.3	1.0	9,704	71.1	9,704	71.1	1.0
Rural	7,685	27.6	7,685	27.6		3,761	26.6	3,761	26.6		3,924	28.7	3,924	28.7	
Not known	52	0.2	52	0.2		22	0.2	22	0.2		30	0.2	30	0.2	
**Socioeconomic status**															
Most disadvantaged	5,852	21.1	5,852	21.1	1.0	3,132	22.2	3,132	22.2	1.0	2,720	19.9	2,720	19.9	1.0
2	6,533	23.5	6,533	23.5		3,320	23.5	3,320	23.5		3,213	23.5	3,213	23.5	
3	6,037	21.7	6,037	21.7		3,065	21.7	3,065	21.7		2,972	21.8	2,972	21.8	
4	3,028	10.9	3,028	10.9		1,572	11.1	1,572	11.1		1,456	10.7	1,456	10.7	
Least disadvantaged	6,297	22.7	6,297	22.7		3,031	21.4	3,031	21.4		3,266	23.9	3,266	23.9	
Not known	54	0.2	54	0.2		23	0.2	23	0.2		31	0.2	31	0.2	
**Number of other health conditions**															
0	25,829	92.9	27,405	98.6	< 0.0001	12,989	91.8	13,966	98.8	< 0.0001	12,840	94.0	13,439	98.4	< 0.0001
≥ 1	1,972	7.1	396	1.4		1,154	8.2	177	1.3		818	6.0	219	1.6	
**Co-occurring disorders**															
1 disorder	24,417	87.8	-	-	-	12,706	89.8	-	-	-	11,711	85.7	-	-	-
≥ 2 disorders	3,384	12.2	-	-		1,437	10.2	-	-		1,947	14.3	-	-	

Health service use post the index admission (in terms of ED visits, hospital admissions, and ambulatory mental health service contacts) was higher for young males and females with a mental disorder than their matched peers. Young males with a mental disorder had a three times higher proportion (57.7% vs. 18.6%, respectively), and young females with a mental disorder had a three and a half times higher proportion (73.5% vs. 39.0%, respectively), of having further hospital admissions after their index admission than their peers (Table 2). Mental and behavioural disorders accounted for 20.3% of readmissions for males and 28.3% for females who were hospitalised with a mental disorder (Supplementary Table 3).

**Table 2:** Further emergency department visits, hospital admissions and ambulatory mental health service contacts for young people hospitalised with a mental disorder and their matched comparison by sex, linked health and mortality data NSW, 2005–2018

	All persons					Male					Female				
	**Case** (*n* = 27,801)		**Comparison** (*n* = 27,801)			**Case** (*n* = 14,143)		**Comparison** (*n* = 14,143)			**Case** (*n* = 13,658)		**Comparison** (*n* = 13,658)		
**Health service use**	**n**	**%**	**n**	**%**	***p*-value**	**n**	**%**	**n**	**%**	***p*-value**	**n**	**%**	**n**	**%**	***p*-value**
**Emergency department visits post the index admission**															
No ED visits	7,614	27.4	15,773	56.7	< 0.0001	3,993	28.2	7,446	52.7	< 0.0001	3,621	26.5	8,327	61.0	< 0.0001
1–2 ED visit	7,894	28.4	7,527	27.1		4,215	29.8	4,120	29.1		3,679	26.9	3,407	25.0	
3–4 ED visits	4,195	15.1	2,464	8.9		2,190	15.5	1,396	9.9		2,005	14.7	1,068	7.8	
≥ 5 ED visits	8,098	29.1	2,037	7.3		3,745	26.5	1,181	8.4		4,353	31.9	856	6.3	
Mean number of visits (SD)	4.5	(8.5)	1.2	(2.5)	< 0.0001	4.0	(7.1)	1.7	(2.9)	< 0.0001	5.0	(9.8)	1.5	(3.0)	< 0.0001
**Hospital admissions post the index admission**															
No admissions	11,674	42.0	22,774	84.9	< 0.0001	6,196	43.8	11,518	81.4	< 0.0001	5,478	40.1	11,256	82.4	< 0.0001
1–2 admission	8,849	31.8	4,419	15.9		4,561	32.3	2,346	16.6		4,288	31.4	2,073	15.2	
3–4 admissions	3,056	11.0	450	1.6		1,431	10.1	214	1.5		1,625	11.9	236	1.7	
≥ 5 admissions	4,222	15.2	158	0.6		1,955	13.8	65	0.5		2,267	16.6	93	0.7	
Mean number of admissions (SD)	3.0	(9.8)	0.3	(1.3)	< 0.0001	3.0	(10.4)	0.5	(1.3)	< 0.0001	3.1	(9.2)	0.6	(2.0)	< 0.0001
**Hospital length of stay**, cumulative post the index admission (days)															
None	11,156	40.1	22,774	81.9	< 0.0001	5,978	42.3	11,518	81.4	< 0.0001	5,178	37.9	11,256	82.4	< 0.0001
1–2	5,517	19.8	3,602	13.0		3,115	22.0	1,949	13.8		2,402	17.6	1,653	12.1	
3–4	2,326	8.4	726	2.6		1,228	8.7	365	2.6		1,098	8.0	361	2.6	
5–7	1,823	6.7	358	1.3		877	6.2	166	1.2		946	6.9	192	1.4	
≥ 8	6,979	25.1	341	1.2		2,945	20.8	145	1.0		4,034	29.5	196	1.4	
Mean hospital cumulative LOS (SD)	15.7	(61.0)	0.6	(3.4)	< 0.0001	12.7	(56.8)	0.9	(4.4)	< 0.0001	18.8	(64.9)	1.1	(4.7)	< 0.0001
**Ambulatory mental health service contacts**															
None	15,373	55.3	26,821	96.5	< 0.0001	9,205	65.1	13,670	96.7	< 0.0001	6,168	45.2	13,151	96.3	< 0.0001
1–2	2,006	7.2	285	1.0		953	6.7	143	1.0		1,053	7.7	142	1.0	
3–4	2,161	7.8	225	0.8		874	6.2	117	0.8		1,287	9.4	108	0.8	
5–7	2,667	10.0	185	0.7		1,027	7.3	94	0.7		1,640	12.0	91	0.7	
≥ 8	5,594	20.1	285	1.0		2,084	14.7	119	0.8		3,510	25.7	166	1.2	
Mean number of contacts (SD)	1.3	(1.7)	0.1	(0.5)	< 0.0001	1.0	(1.5)	0.1	(0.5)	< 0.0001	1.7	(1.7)	0.1	(0.6)	< 0.0001

**Fig. 1:**
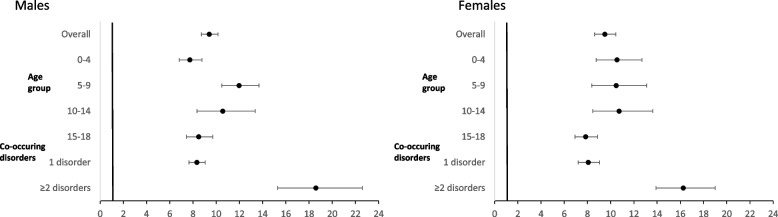
Adjusted rate ratio of further hospital admissions for young people hospitalised with a mental disorder and their matched comparison by sex, linked health and mortality data NSW, 2005–2018^1^. ^1^Adjusted for sex, age group, comorbidities (Y/N), location of residence, and socioeconomic status.  Excludes *n*=54 missing location of residence/socioeconomic status

After adjusting for covariates, both young males (ARR 9.40; 95%CI 8.72–10.15) and females (ARR 9.49; 95%CI 8.62–10.44) with a mental disorder had a higher risk of further hospitalisations than their matched peers. The risk of admission was highest for males aged 5–9 years (ARR 11.97; 95%CI 10.47–13.69) and females aged 10–14 years (ARR 10.73; 95%CI 8.45–13.62) compared to matched counterparts. Compared to matched peers, young males (ARR 18.59; 95%CI 15.29–22.60) and females (ARR 16.25; 95%CI 13.90-19.01) with ≥ 2 co-occurring mental disorders had double the risk of admission compared to young males (ARR 8.33; 95%CI 7.66–9.06) and females (ARR 8.07; 95%CI 7.20–9.04) with one disorder, respectively (Fig. 1 and Supplementary Table 4).

**Fig. 2:**
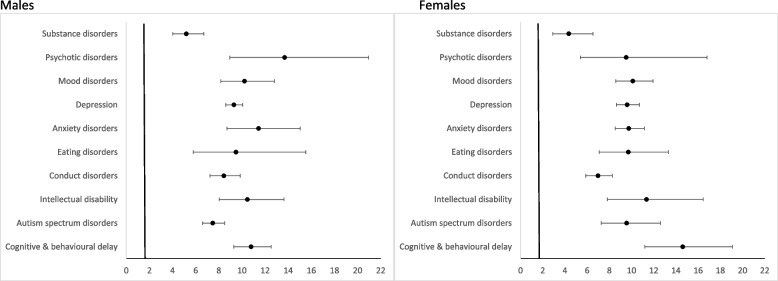
Adjusted rate ratio of further hospital admissions for young people hospitalised with a mental disorder by sex and disorder type and their matched comparison, linked health and mortality data NSW, 2005–2018^1−2^. ^1^Adjusted for sex, age group, comorbidities (Y/N), location of residence, and socioeconomic status.  Excludes *n*=54 missing location of residence/socioeconomic status. ^2^For psychotic disorders the reference age group was 5-9 years as no young people with a psychotic disorder were aged 0-4 years

After disaggregating by disorder type at index admission, young people with each type of disorder had a higher risk of further hospitalisations than their matched peers. Young males with psychotic disorders (ARR 13.69; 95%CI 8.95–20.94), anxiety disorders (ARR 11.44; 95%CI 8.70-15.04), cognitive and behavioural delays (ARR 10.79; 95%CI 9.30-12.53), intellectual disability (ARR 10.47; 95%CI 8.04–13.64), and mood disorders (ARR 10.23; 95%CI 8.17–12.80) had more than a 10-fold higher risk of further hospital admissions compared to matched peers. Young females with cognitive and behavioural delays (ARR 14.62; 95%CI 11.20-19.08), intellectual disability (ARR 11.35; 95%CI 7.83–16.45), and mood disorders (ARR 10.12; 95%CI 8.58–11.93), had a 10-fold higher risk of further hospitalisations compared to matched peers (Fig. 2 and Supplementary Table 5).

## Discussion

This study identified that young people hospitalised with a mental disorder of both sexes had a higher risk of further hospital admission for either their mental disorder or other physical conditions than their matched peers during the study period. The risk was over 10-fold higher for males with psychotic and anxiety disorders, and was higher for both males and females with cognitive and behavioural delays, intellectual disability, and mood disorders compared to peers. Young people with ≥ 2 co-occurring mental disorders had the highest risk of further hospitalisations compared to their matched counterparts.

Hospital admissions of young people with mental disorders are increasing in a number of high-income countries [15, 25, 26]. This increase is at least partly due to increasing awareness and diagnosis of mental disorders in young people, along with a potential increase in risk factors for young people, including poor psychological health, genetic vulnerability stemming from a family psychiatric history, and adverse environmental conditions and interactions [25]. In the US, mental disorders were the most commonly given reason for hospital admissions of young person who had existing health conditions [27]. Young people with a mental disorder in the US were also more likely to have multiple hospital visits compared to young people hospitalised for other health conditions [15].

In the current study young males and females diagnosed with psychotic disorders had a 13- and 9-fold increased probability of further hospital admissions than their matched counterparts, respectively. This is consistent with the findings of a recent systematic review and meta-analysis, which identified that psychotic disorders were associated with repeat hospitalisations among adolescents [28]. Similarly, psychosis was the most common principal diagnosis identified in a New Zealand study of admissions to a child and adolescent psychiatric inpatient unit [26]. However, the frequency of hospital admissions for psychotic disorders was not only related to the course of the illness itself, but can be influenced by factors such as adverse childhood circumstances, mental disorder among carers, and ongoing substance use, particularly the use of cannabis [26, 29].

Young people with an intellectual disability were identified as having more than a 10-fold higher risk of further hospitalisation than their peers, consistent with the other studies [30]. More frequent use of hospital services are due to both the behavioural disturbances associated with many forms of intellectual disability, and the existence of a range of comorbid physical disabilities and comorbidities compared to peers [31]. People with an intellectual disability can experience barriers to accessing primary health care, which can lead to their increased use of hospital services [31]. In fact, preventable hospital admissions are known to be more common among people with an intellectual disability of all ages compared to the general population [32].

This study found a seven- and nine-fold higher risk of further hospitalisation for both males and females with ASD compared to peers, respectively. This finding is consistent with studies elsewhere that found young people with ASDs were twice as likely to be hospitalised than the general population [33]. Young people with ASD can experience difficulties in social interactions, sensory processing, behaviour and communication, and often require treatment for co-occurring psychological comorbidities (commonly attention deficit hyperactivity disorder, anxiety and depression) and physical illness [33, 34] that can all contribute to their high healthcare needs and utilisation. Children with ASDs aged 1–8 years can experience a higher proportion of comorbidities than their peers and have a higher likelihood of being admitted to hospital and a longer hospital LOS than children without ASD [35]. Young people with ASDs can also experience difficulties in accessing primary care and report negative experiences with healthcare providers [34, 36].

In the current study, young people diagnosed with mood or anxiety disorders had a ten-fold higher risk of a further hospital admission than their counterparts. Depression among young people aged 13–17 years has been associated with increased healthcare use and associated treatment cost in the US [37]. In a Californian study of young people with a mental disorder aged 5–17 years, the most common reasons for admission were depression (27%) and anxiety-related disorders (14%) [38], possibly because of the higher incidence of self-harm associated with these disorders, as young people who self-harm or express suicidal ideas have at least twice the risk of hospitalisation than those without suicidal ideation [28].

Some young people are reluctant to seek mental health care for reasons such as the stigma associated with mental illness and poor mental health literacy [39, 40]. The accessibility, cost and availability of specialist services may also present a significant impediment to adequate mental health support for young people [39]. Moreover, mental disorders experienced by young people are often closely linked to their family environments, social circumstances and life events, which are not easily modified by healthcare interventions and may require other preventive and supportive measures, such as educational interventions [41]. Nevertheless, early detection and timely access to mental health services are important [17], as successful intervention for treatable mental disorders can have a lasting effect on the trajectory of a young person’s future mental health, social and educational performance. Promising interventions can involve symptom management, encouraging adherence to treatment regimes, developing a supportive environment and social network, and better access to primary care and specialist mental health services [29, 42–44], which, along with early detection and timely access to mental health services [17], are important and are likely to strengthen a young person’s capacity to manage adverse situations.

Hospital admissions for treatment of mental disorders in young people are influenced by a number of factors, including both the availability of specialist hospital beds [25], hospital admission policies, and also the availability of community-based alternatives and care pathways [31]. In 2018-19, the majority of public hospital beds allocated for mental health admissions in Australia were for general adult services (71.4%; 5,002 beds), with only 4.3% (303 beds) allocated for child and adolescent services [45]. A further 1.0% (70 beds) were allocated for youth services in Australia in 2011-12 [45].

Further research could explore the type of co-occurring health conditions experienced by hospitalised young people with a mental disorder, as well as examining their health service use trajectories over time, including reasons for repeated readmissions and identification of factors influencing the frequency of health service use. The transition from paediatric to adult mental health services is an area of particular concern [46], and the effect of interventions in adolescence on later health service use is an area that deserves further investigation. A better understanding of unmet health service needs of young people with a mental disorder should be identified, as up to 38% of young people with a mental disorder indicated they had not sought or received treatment for their conditions from health professionals [39].

The strength of this study was that it was a large population-based study linking birth, ED visit, hospital admission, ambulatory contacts and mortality records over a 13-year period. However, there were some study limitations. In most cases, only comorbidities relevant to the admission are indicated in hospital diagnosis classifications, therefore it is possible that some comorbidities experienced by young people were not recorded. This is particularly likely for the comparison cohort, where not all had been admitted to hospital and, as a result, there was no opportunity to identify comorbidities, despite the three-year lookback period. The count of hospital readmissions was not disaggregated by mental and physical conditions, but as indicated in Supplementary Tables 3, mental disorders accounts for 20.3–28.3% of readmissions for young males and females, respectively. No assessment of data validity was able to be conducted and it is possible that there could be some data misclassification. A small number of residential postcodes could not be identified, which affected socioeconomic and regional classification of those cases.

The study only compared young people who had been hospitalised for a mental disorder, and did not include young people presenting solely to mental health professionals in private practice for treatment. In Australia, the threshold for hospitalisation for young people with a mental disorder is high, as there are comparatively few mental health inpatient beds for this cohort, and the main reasons for admission are concern for a young person’s safety, or for diagnostic clarification or treatment of a condition. Hence, hospitalised young people with a mental disorder in Australia are likely to be the most seriously affected, regardless of diagnosis and irrespective of the number of days spent in hospital. Information on visits to private hospital EDs were not available and information on ambulatory mental health contacts at public hospitals were only available from 2006.

## Conclusion

This study identified that young males and females with a mental disorder had a higher risk of further hospitalisation than their peers. These findings contribute to the understanding of health service use among young people with a mental disorder, support the need for the development of community and hospital-based services that both prevent unnecessary hospital admissions in childhood and adolescence that can potentially reduce the burden and loss arising from mental disorders in adult life.

## Supplementary Information

**Additional file 1:** **Supplementary Table 1.** Case identification and diagnostic classification. **Supplementary Table 2.** Health conditions and ICD-10-AM classifications. **Supplementary Table 3.** Principal diagnosis of the first read mission for young people hospitalised with a mental disorder by sex, linked health and mortality data NSW, 2005-2018. **Supplementary Table 4.** Rate ratio of further hospital admissions for young people hospitalised with a mental disorder and their matched comparison by sex, linked health and mortality data NSW, 2005-2018. **Supplementary Table 5.** Rate ratio of further hospital admissions for young people hospitalised with a mental disorder by sex and disorder type and their matched comparison, linked health and mortality data NSW, 2005-2018.

## Data Availability

The datasets analysed during the current study are not publicly available as they were used under licence. The data that support the findings of this study are available from the NSW Health Department via submitting an application for access to the data via MOH-CHeReL@health.nsw.gov.au and via following the requirements specified at https://www.cherel.org.au/apply-for-linked-data.

## References

[1] World Health Organization. Adolescent mental health. 2021 [cited 2021 17/11/2021]; Available from: https://www.who.int/news-room/fact-sheets/detail/adolescent-mental-health.

[2] Bardach NS, Coker TR, Zima BT, Murphy JM, Knapp P, Richardson LP (2014). Common and costly hospitalizations for pediatric mental health disorders. Pediatrics..

[3] Hoffmann JA, Stack AM, Samnaliev M, Monuteaux MC, Lee LK (2019). Trends in visits and costs for mental health emergencies in a pediatric emergency department, 2010–2016. Acad Pediatr.

[4] Sadler K, Vizard T, Ford T, Marchesell F, Pearce N, Mandalia D (2018). Mental health of children and young people in England, 2017.

[5] Lawrence D, Hafekost J, Johnson SE, Saw S, Buckingham WJ, Sawyer MG (2016). Key findings from the second Australian child and adolescent survey of mental health and wellbeing. Australian & New Zealand Journal of Psychiatry.

[6] Allerton LA, Welch V, Emerson E (2011). Health inequalities experienced by children and young people with intellectual disabilities: a review of literature from the United Kingdom. J Intellect Disabil.

[7] Iacono T, Bigby C, Unsworth C, Douglas J, Fitzpatrick P (2014). A systematic review of hospital experiences of people with intellectual disability. BMC Health Serv Res.

[8] Patel V, Flisher AJ, Hetrick S, McGorry P (2007). Mental health of young people: a global public-health challenge. The Lancet.

[9] Olusunmade M, Qadir T, Akyar S, Farid A, Aggarwal R (2019). Incremental hospital utilization and mortality associated with co-morbid depression in pediatric hospitalizations. J Affect Disord.

[10] Bowman S, McKinstry C, McGorry P (2017). Youth mental ill health and secondary school completion in Australia: time to act. Early Interv Psychiat.

[11] Leach LS, Butterworth P (2012). The effect of early onset common mental disorders on educational attainment in Australia. Psychiatry Res.

[12] Mental Health Commission. National Children’s Mental Health and Wellbeing Strategy. Canberra; 2021.

[13] Mitchell RJ, McMaugh A, Schniering C, Cameron CM, Lystad RP, Badgery-Parker T, Nielssen O. Mental disorders and their impact on school performance and high school completion by gender in Australia: A matched population-based cohort study. Aust N Z J Psychiatry, 2021:online first. 10.1177/00048674211061684 34875885

[14] Fleming M, Fitton CA, Steiner MF, McLay JS, Clark D, King A (2020). Educational and health outcomes of children and adolescents receiving antidepressant medication: Scotland-wide retrospective record linkage cohort study of 766 237 schoolchildren. Int J Epidemiol.

[15] Torio CM, Encinosa W, Berdahl T, McCormick MC, Simpson LA (2015). Annual report on health care for children and youth in the United States: national estimates of cost, utilization and expenditures for children with mental health conditions. Acad Pediatr..

[16] Mapelli E, Black T, Doan Q (2015). Trends in pediatric emergency department utilization for mental health-related visits. J Pediatr.

[17] Segal L, Guy S, Furber G (2018). What is the current level of mental health service delivery and expenditure on infants, children, adolescents, and young people in Australia?. Australian & New Zealand Journal of Psychiatry.

[18] Mitchell R, Cameron C, Lystad R, Nielssen O, McMaugh A, Herkes G (2019). Impact of chronic health conditions and injury on school performance and health outcomes in New South Wales, Australia: a study protocol. BMJ Paediatrics.

[19] Miller C, Shi J, Wheeler K, Yin H, Smith GA, Groner J, Xiang H (2013). Chronic conditions and outcomes of pediatric trauma patients. J Trauma Acute Care.

[20] Edwards J, Houtrow A, Vasilevskis E, Rehm R, Markovitz B, Graham R, Dudley A (2012). Chronic conditions among children admitted to US pediatric intensive care units: Their prevalence and impact on risk for mortality and prolonged length of stay. Crit Care Med.

[21] Mitchell R, Curtis K, Braithwaite J (2017). Health outcomes and costs for injured young people hospitalised with and without chronic health conditions. Injury.

[22] Australian Bureau of Statistics (2011). Census of Population and Housing: Socio-Economic Indexes for Areas (SEIFA), Australia. Catalogue no: 2033.0.55.001.

[23] Australian Bureau of Statistics. 1270.0.55.005 - Australian Statistical Geography Standard (ASGS): Volume 5 - Remoteness Structure, July 2011. 2013 23/07/2014 [cited 2014 03/09/2014]; Available from: http://www.abs.gov.au/AUSSTATS/abs@.nsf/DetailsPage/1270.055.005July%202011?OpenDocument.

[24] Pearce N (2016). Analysis of matched case-control studies. BMJ.

[25] Zanato S, Miscioscia M, Traverso A, Gatto M, Poli M, Raffagnato A, Gatta M. A Retrospective Study on the Factors Associated with Long-Stay Hospitalization in a Child Neuropsychiatry Unit. In: Healthcare. Canberra: Multidisciplinary Digital Publishing Institute; 2021. 10.3390/healthcare9091241 PMC8465245 34575015

[26] van Kessel K, Myers E, Stanley S, Reed LW (2012). Trends in child and adolescent discharges at a New Zealand psychiatric inpatient unit between 1998 and 2007. NZ Med J.

[27] Berry JG, Ash AS, Cohen E, Hasan F, Feudtner C, Hall M (2017). Contributions of children with multiple chronic conditions to pediatric hospitalizations in the United States: a retrospective cohort analysis. Hosp Pediatr.

[28] Edgcomb JB, Sorter M, Lorberg B, Zima BT (2020). Psychiatric readmission of children and adolescents: a systematic review and meta-analysis. Psychiatric Serv.

[29] Paruk S, Ramlall S, Burns J (2009). Adolescent-onset psychosis: A 2-year retrospective study of adolescents admitted to a general psychiatric unit. South Afr J Psychiatry.

[30] Kim J, Stevens P, Carbone PS, Jones KB (2020). Health care use and spending of pediatric patients with an intellectual or developmental disability. Med Care.

[31] Bebbington A, Glasson E, Bourke J, De Klerk N, Leonard H (2013). Hospitalisation rates for children with intellectual disability or autism born in Western Australia 1983–1999: a population-based cohort study. BMJ Open.

[32] Weise JC, Srasuebkul P, Trollor JN (2021). Potentially preventable hospitalisations of people with intellectual disability in New South Wales. Med J Aust..

[33] Ames JL, Massolo ML, Davignon MN, Qian Y, Croen LA (2021). Healthcare service utilization and cost among transition-age youth with autism spectrum disorder and other special healthcare needs. Autism.

[34] Beverly J, Giannouchos T, Callaghan T. Examining frequent emergency department use among children and adolescents with autism spectrum disorder. Autism. 2021;25(5). 10.1177/1362361321990925. 10.1177/1362361321990925 33567902

[35] Dizitzer Y, Meiri G, Flusser H, Michaelovski A, Dinstein I, Menashe I. Comorbidity and health services’ usage in children with autism spectrum disorder: A nested case–control study. Epidemiol Psychiatr Sci. 2020;29. 10.1017/S2045796020000050 PMC7214718 31987063

[36] Iannuzzi D, Hall M, Oreskovic NM, Aryee E, Broder-Fingert S, Perrin JM, Kuhlthau KA. Emergency Department Utilization of Adolescents and Young Adults with Autism Spectrum Disorder. J Autism Dev Disord. 2021:1–6. 10.1007/s10803-021-04969-y 33751374

[37] Wright DR, Katon WJ, Ludman E, McCauley E, Oliver M, Lindenbaum J, Richardson LP (2016). Association of adolescent depressive symptoms with health care utilization and payer-incurred expenditures. Acad Pediatr.

[38] Huffman LC, Wang NE, Saynina O, Wren FJ, Wise PH, Horwitz SM (2012). Predictors of hospitalization after an emergency department visit for California youths with psychiatric disorders. Psychiatric Serv.

[39] Gulliver A, Griffiths KM, Christensen H (2010). Perceived barriers and facilitators to mental health help-seeking in young people: a systematic review. BMC Psychiatry.

[40] Reavley NJ, Jorm AF (2011). Young people’s recognition of mental disorders and beliefs about treatment and outcome: findings from an Australian national survey. Australian & New Zealand Journal of Psychiatry.

[41] Yamaguchi S, Ojio Y, Foo JC, Michigami E, Usami S, Fuyama T (2020). A quasi-cluster randomized controlled trial of a classroom-based mental health literacy educational intervention to promote knowledge and help-seeking/helping behavior in adolescents. J Adolesc.

[42] Vander Stoep A, Weiss NS, Kuo ES, Cheney D, Cohen P (2003). What proportion of failure to complete secondary school in the US population is attributable to adolescent psychiatric disorder?. J Behav Health Serv Res.

[43] McIntyre JC, Worsley J, Corcoran R, Harrison Woods P, Bentall RP (2018). Academic and non-academic predictors of student psychological distress: The role of social identity and loneliness. J Mental Health.

[44] Wilson CJ, Deane FP, Marshall KL, Dalley A (2008). Reducing adolescents’ perceived barriers to treatment and increasing help-seeking intentions: effects of classroom presentations by general practitioners. J Youth Adolesc.

[45] Australian Institute of Health (2021). Mental health services in Australia.

[46] Kennedy A, Sawyer S (2008). Transition from pediatric to adult services: are we getting it right?. Curr Opin Pediatr.

