# Supplementary material for: Health service use for young males and females with a mental disorder is higher than their peers in a population-level matched cohort
Source: BMC Health Serv Res. 2022 Nov 16;22:1359. doi: 10.1186/s12913-022-08789-3 (PMC9670362; doi:10.1186/s12913-022-08789-3)
Supplement: Supplementary file 1 — Additional file 1: Supplementary Table 1. Case identification and diagnostic classification. Supplementary Table 2. Health conditions and ICD-10-AM classifications. Supplementary Table 3. Principal diagnosis of the first read mission for young people hospitalised with a mental disorder by sex, linked health and mortality data NSW, 2005-2018. Supplementary Table 4. Rate ratio of further hospital admissions for young people hospitalised with a mental disorder and their matched comparison by sex, linked health and mortality data NSW, 2005-2018. Supplementary Table 5. Rate ratio of further hospital admissions for young people hospitalised with a mental disorder by sex and disorder type and their matched comparison, linked health and mortality data NSW, 2005-2018. [file 12913_2022_8789_MOESM1_ESM.docx]

**Supplementary Table 1: Case identification and diagnostic classification**

| **Mental disorder** | **ICD-10-AM** |
| --- | --- |
| **Substance disorders** (incl. alcohol and drug abuse and dependence) | F10-F19 |
| **Psychotic disorders** (incl. schizophrenia, schizotypal and delusional disorders) | F20-F29 |
| **Mood disorders** (incl. manic episode, bipolar affective disorder, depression, affective disorders) | F30-F39 |
| *Depression (as a subcategory of mood disorders)* | F20.4, F31.3, F31.4, F31.5, F32.0, F32.1, F32.2, F32.3, F32.8, F32.9, F33.0, F33.1, F33.2, F33.3, F33.4, F33.8, F33.9, F34.1, F41.2, F43.2 |
| **Anxiety disorders** (incl. anxiety disorder, phobic anxiety disorders, obsessive-compulsive disorder, stress and adjustment disorders, including post-traumatic stress disorder (PTSD), dissociative disorders, somatoform disorders, other neurotic disorders) | F40-F48 |
| **Eating disorders** (incl. anorexia, bulimia, overeating, vomiting associated with psychological disturbances) | F50 |
| **Intellectual disability** (incl. mild, moderate, severe and profound intellectual disability, other and unspecified intellectual disability) | F70-F79 |
| **Autism spectrum disorders** (incl. childhood and atypical autism, Rhett’s syndrome, other childhood disintegrative disorders, Asperger’s syndrome, other and unspecified pervasive developmental disorders) | F84 |
| **Cognitive and behavioural delay** (incl. speech and language disorders, motor function development disorders, other and unspecified disorders of psychological development) | F80-F83 and F88-F89 |
| **Conduct disorders** (incl. hyperkinetic disorders, conduct disorders, mixed disorders of conduct and emotions, emotional disorders and social functioning disorders with onset specific to childhood and adolescence, tic disorders) | F90-F98 |

Supplementary Table 2: Health conditions and ICD-10-AM classifications

| **Health condition** | **ICD-10-AM classifications** |
| --- | --- |
| **Circulatory system** |  |
| Hypertension | I10-I15 |
| **Congenital malformations** |  |
| All congenital malformations | Q00-Q99 |
| *Congenital malformation of the heart and great arteries* | Q20-Q25 |
| **Digestive system and allergies** |  |
| Celiac disease and other serious allergies | K52.2, K90.0, T78.0, T78.2, T78.4 |
| **Endocrine, nutritional and metabolic conditions** |  |
| Diabetes | E09-E14 |
| Obesity | E66 |
| Cystic fibrosis | E84 |
| **Immune system conditions and coagulation defects** |  |
| Anaemia | D50-D53 and D55-D64 |
| Coagulation defects (e.g. haemophilia) | D65-C68 |
| **Mental health conditions** |  |
| Autism spectrum disorders | F84 |
| Behavioural and emotional disorders of childhood | F90-F98 |
| Cognitive and behavioural delay | F80-F83 and F88-F89 |
| Eating disorders | F50 |
| Hyperkinetic disorder | F90 |
| Mental retardation | F70-F79 |
| Mood affective disorders | F30-F39 |
| Neurotic, stress-related and somatoform disorders | F40-F48 |
| Personality disorders | F60-F69 |
| Schizophrenia, schizotypal and delusions disorders | F20-F29 |
| **Neoplasms** |  |
| All malignancies | C00-D48 |
| *Acute lymphoblastic leukaemia and acute myeloid leukaemia* | C91.0, C92.0 |
| *Brain cancer* | C71 |
| **Nervous system conditions** |  |
| Cerebral palsy | G80 |
| Epilepsy | G40, G41 |
| **Renal conditions** | I12.0, I13.1, NO3, NO5, N18-N19, N25.0, Z49, Z94.0, Z99.2 |
| **Respiratory conditions** |  |
| Chronic lower respiratory disease | J40-J47 |
| *Asthma* | J45 |

Supplementary Table 3: Principal diagnosis of the first readmission for young people hospitalised with a mental disorder by sex, linked health and mortality data NSW, 2005-2018

|  | **Males**  (n=7,877) | | **Females**  (n=8,090) | |
| --- | --- | --- | --- | --- |
|  | **n** | **%** | **n** | **%** |
| Certain infectious and parasitic diseases | 268 | 3.4 | 207 | 2.6 |
| Neoplasms | 100 | 1.3 | 77 | 1.0 |
| Diseases of the blood and blood-forming organs and certain disorders involving the immune mechanism | 41 | 0.5 | 29 | 0.4 |
| Endocrine, nutritional and metabolic diseases | 121 | 1.5 | 125 | 1.5 |
| Mental and behavioural disorders | 1,600 | 20.3 | 2,288 | 28.3 |
| Diseases of the nervous system | 584 | 7.4 | 379 | 4.7 |
| Diseases of the eye and adnexa | 91 | 1.2 | 54 | 0.7 |
| Diseases of the ear and mastoid process | 365 | 4.6 | 215 | 2.7 |
| Diseases of the circulatory system | 51 | 0.6 | 46 | 0.6 |
| Disease of the respiratory system | 754 | 9.6 | 538 | 6.7 |
| Diseases of the digestive system | 1,000 | 12.7 | 665 | 8.2 |
| Diseases of the skin and subcutaneous tissue | 189 | 2.4 | 100 | 1.2 |
| Diseases of the musculoskeletal system and connective tissue | 153 | 1.9 | 136 | 1.7 |
| Diseases of the genitourinary system | 172 | 2.2 | 268 | 3.3 |
| Pregnancy, childbirth and the puerperium | 0 | 0.0 | 372 | 4.6 |
| Injury, poisoning and certain other consequences of external causes | 979 | 12.4 | 1162 | 14.4 |
| Factors influencing health status and contact with health services | 343 | 4.4 | 241 | 3.0 |
| Other diagnoses | 1,066 | 13.5 | 1,188 | 14.7 |

**Supplementary Table 4: Rate ratio of further hospital admissions for young people hospitalised with a mental disorder and their matched comparison by sex, linked health and mortality data NSW, 2005-2018**

|  | **All persons** | | | | **Male** | | | | **Female** | | | |
| --- | --- | --- | --- | --- | --- | --- | --- | --- | --- | --- | --- | --- |
| **Characteristic** | **Unadjusted rate ratio** | **95% CIs** | **Adjusted**^1^ **rate ratio** | **95% CIs** | **Unadjusted rate ratio** | **95% CIs** | **Adjusted rate ratio** | **95% CIs** | **Unadjusted rate ratio** | **95% CIs** | **Adjusted rate ratio** | **95% CIs** |
| **Overall** | 10.30* | 9.67-10.97 | 9.43* | 8.87-10.03 | 10.40* | 9.62-11.24 | 9.40* | 8.72-10.15 | 10.12* | 9.19-11.15 | 9.49* | 8.62-10.44 |
| **Age group** |  |  |  |  |  |  |  |  |  |  |  |  |
| 0-4 | 9.96* | 9.00-11.01 | 8.67* | 7.81-9.62 | 8.84* | 7.80-10.00 | 7.73* | 6.81-8.78 | 12.21* | 10.24-14.57 | 10.54* | 8.76-12.70 |
| 5-9 | 13.65* | 12.06-15.46 | 11.53* | 10.27-12.94 | 14.12* | 12.15-16.42 | 11.97* | 10.47-13.69 | 12.62* | 10.14-15.71 | 10.47* | 8.37-13.09 |
| 10-14 | 11.22* | 9.40-13.39 | 10.65* | 8.92-12.70 | 11.76* | 9.25-14.95 | 10.56* | 8.35-13.36 | 10.88* | 8.57-13.81 | 10.73* | 8.45-13.62 |
| 15-18 | 8.22* | 7.39-9.14 | 8.02* | 7.31-8.81 | 8.78* | 7.69-10.03 | 8.49* | 7.43-9.70 | 8.04* | 7.01-9.22 | 7.84* | 6.93-8.86 |
| **Co-occurring disorders** |  |  |  |  |  |  |  |  |  |  |  |  |
| 1 disorder | 9.05* | 8.42-9.73 | 8.19* | 7.63-8.79 | 9.31* | 8.55-10.14 | 8.33* | 7.66-9.06 | 8.75* | 7.79-9.81 | 8.07* | 7.20-9.04 |
| ≥2 disorders | 18.19* | 16.08-20.58 | 17.14* | 15.14-19.41 | 20.04* | 16.48-24.38 | 18.59* | 15.29-22.60 | 16.95* | 14.49-19.83 | 16.25* | 13.90-19.01 |

^*^p<0.0001.

^1^Adjusted for sex, age group, comorbidities (Y/N), location of residence, and socioeconomic status. Excludes n=54 missing location of residence/socioeconomic status.

**Supplementary Table 5: Rate ratio of further hospital admissions for young people hospitalised with a mental disorder by sex and disorder type and their matched comparison, linked health and mortality data NSW, 2005-2018**

|  |  |  | **Unadjusted rate ratio** | | **Adjusted rate ratio^1^** | |
| --- | --- | --- | --- | --- | --- | --- |
| **Characteristic** | **n** | **%** | **RR** | **95%CI** | **ARR**^1^ | **95%CI** |
| **All persons** |  |  |  |  |  |  |
| ***Type of disorder at index admission*** |  |  |  |  |  |  |
| Substance disorders | 2,436 | 8.8 | 4.46* | 3.25-6.10 | 4.64* | 3.54-6.08 |
| Psychotic disorders^2^ | 443 | 1.6 | 11.04* | 7.58-16.10 | 11.22* | 7.77-16.20 |
| Mood disorders | 3,711 | 13.4 | 10.28* | 8.92-11.85 | 10.18* | 8.89-11.67 |
| *Depression* | *2,807* | *10.1* | *10.38** | *9.70-11.11* | *9.44** | *8.83-10.09* |
| Anxiety disorders | 5,989 | 21.5 | 11.25* | 9.75-12.99 | 10.26* | 9.03-11.66 |
| Eating disorders | 1,272 | 4.6 | 10.01* | 7.38-13.78 | 9.90* | 7.39-13.26 |
| Conduct disorders | 4,932 | 17.7 | 8.75* | 7.69-9.96 | 7.95* | 7.06-8.96 |
| Intellectual disability | 1,075 | 3.9 | 13.62* | 10.52-17.63 | 10.74* | 8.51-13.57 |
| Autism spectrum disorders | 3,696 | 13.3 | 8.78* | 7.79-9.89 | 7.92* | 7.05-8.90 |
| Cognitive & behavioural delay | 4,247 | 15.3 | 14.34* | 12.59-16.34 | 12.27* | 10.69-14.09 |
| **Male** |  |  |  |  |  |  |
| ***Type of disorder at index admission*** |  |  |  |  |  |  |
| Substance disorders | 1,343 | 9.5 | 4.91* | 3.70-6.50 | 5.19* | 4.02-6.71 |
| Psychotic disorders^2^ | 259 | 1.8 | 14.09* | 9.23-21.50 | 13.69* | 8.95-20.94 |
| Mood disorders | 1,053 | 7.5 | 10.75* | 8.54-13.52 | 10.23* | 8.17-12.80 |
| *Depression* | *798* | *5.6* | *10.31** | *9.51-11.17* | *9.31** | *8.61-10.07* |
| Anxiety disorders | 2,104 | 14.9 | 13.31* | 9.90-17.89 | 11.44* | 8.70-15.04 |
| Eating disorders | 146 | 1.0 | 13.75* | 7.82-24.19 | 9.48* | 5.79-15.52 |
| Conduct disorders | 3,087 | 21.8 | 9.23* | 7.75-11.00 | 8.44* | 7.24-9.84 |
| Intellectual disability | 637 | 4.5 | 14.04* | 10.27-19.21 | 10.47* | 8.04-13.64 |
| Autism spectrum disorders | 2,928 | 20.7 | 8.10* | 7.15-9.19 | 7.48* | 6.59-8.49 |
| Cognitive & behavioural delay | 2,586 | 18.3 | 12.99* | 11.26-14.99 | 10.79* | 9.30-12.53 |
| **Female** |  |  |  |  |  |  |
| ***Type of disorder at index admission*** |  |  |  |  |  |  |
| Substance disorders | 1,093 | 8.0 | 4.17* | 2.54-6.83 | 4.35* | 2.90-6.52 |
| Psychotic disorders^2^ | 184 | 1.4 | 9.00* | 5.08-15.94 | 9.52* | 5.39-16.80 |
| Mood disorders | 2,658 | 19.5 | 10.16* | 8.56-12.05 | 10.12* | 8.58-11.93 |
| *Depression* | *2,009* | *14.7* | *10.38** | *9.30-11.57* | *9.61** | *8.63-10.71* |
| Anxiety disorders | 3,885 | 28.4 | 10.54* | 8.87-12.32 | 9.75* | 8.52-11.15 |
| Eating disorders | 1,126 | 8.3 | 9.75* | 6.98-13.62 | 9.71* | 7.08-13.33 |
| Conduct disorders | 1,845 | 13.5 | 7.84* | 6.59-9.33 | 6.98* | 5.89-8.27 |
| Intellectual disability | 438 | 3.2 | 13.09* | 8.54-20.05 | 11.35* | 7.83-16.45 |
| Autism spectrum disorders | 768 | 5.6 | 11.10* | 8.37-14.73 | 9.56* | 7.26-12.61 |
| Cognitive & behavioural delay | 1,661 | 12.2 | 16.50* | 12.85-21.19 | 14.62* | 11.20-19.08 |

*p<0.0001.

^1^Adjusted for sex, age group, comorbidities (Y/N), location of residence, and socioeconomic status. Excludes n=54 missing location of residence/socioeconomic status.

^2^For psychotic disorders the reference age group was 5-9 years as no young people with a psychotic disorder were aged 0-4 years.
